# Electromechanical window as an integrative marker of remodeling and arrhythmic risk in biventricular vs. left bundle branch area pacing

**DOI:** 10.1186/s43044-026-00772-1

**Published:** 2026-08-05

**Authors:** Arvind Kumar, Tania Bansal, Salil Jaura

**Affiliations:** 1 Pragma Medical Institute, Bathinda, India; 2 Adesh Institute of Medical Sciences and Research, Bathinda, India

**Keywords:** Electromechanical window, Cardiac resynchronization therapy, Left bundle branch area pacing, Ventricular remodelling, Arrhythmia

## Abstract

**Background:**

The electromechanical window (EMW), defined as the difference between mechanical and electrical systole, is an emerging marker of arrhythmic risk and ventricular dysfunction. Although cardiac resynchronization therapy (CRT) improves mechanical synchrony, the influence of different pacing strategies on EMW is not well established. This study compared the effects of conventional biventricular (BiV) pacing and left bundle branch area pacing (LBBAP) on EMW, reverse remodeling, CRT response, and arrhythmic burden.

**Methods:**

In this prospective, observational study, 80 patients with left ventricular ejection fraction (LVEF) ≤ 35% undergoing CRT received BiV pacing (*n* = 40) or LBBAP (*n* = 40). Echocardiography with simultaneous electrocardiography was performed at baseline and 6 months. The primary endpoint was change in EMW.

**Results:**

At 6 months, LBBAP was associated with greater QRS narrowing (42 ± 15 vs. 28 ± 13 ms, *p* < 0.001) and EMW normalization (− 38 ± 22 to − 5 ± 18 ms vs. − 36 ± 20 to − 18 ± 19 ms; *p* = 0.003). Improvements in LVEF, global longitudinal strain, and left ventricular end-systolic volume were also greater with LBBAP. EMW improvement correlated with reverse remodeling and QRS narrowing. Baseline EMW predicted CRT response (AUC 0.71), while persistent EMW negativity was associated with a higher incidence of non-sustained ventricular tachycardia (45% vs. 18%, *p* = 0.01).

**Conclusions:**

LBBAP was associated with greater EMW normalization, improved reverse remodeling, and a lower arrhythmic burden than BiV pacing. EMW may represent an integrative marker of CRT response and residual arrhythmic risk. These findings are hypothesis-generating and require validation in larger multicenter randomized studies.

## Introduction

Cardiac resynchronization therapy (CRT) is an established treatment for patients with heart failure with reduced ejection fraction (HFrEF), prolonged QRS duration, and persistent symptoms despite guideline-directed medical therapy. Conventional biventricular (BiV) pacing improves ventricular synchrony, promotes reverse remodeling, and reduces heart failure hospitalization and mortality. Nevertheless, approximately 30% of patients demonstrate limited echocardiographic or clinical response, highlighting the need for improved methods of patient selection and more physiological pacing strategies [1–3].

Left bundle branch area pacing (LBBAP) has emerged as an alternative CRT strategy that directly recruits the His–Purkinje conduction system, resulting in more physiological ventricular activation. Observational studies and multicenter registries have consistently demonstrated narrower paced QRS complexes, improved ventricular function, and favorable clinical outcomes with LBBAP [4–6]. However, recent randomized controlled trials have reported more nuanced findings, suggesting that while LBBAP is generally non-inferior and may provide superior electrical resynchronization and reverse remodeling in selected patients, its clinical benefit depends largely on successful conduction system capture and appropriate patient selection [7–10]. Consequently, the relative advantages of LBBAP over conventional BiV pacing remain an area of active investigation.

Assessment of CRT response has traditionally relied on QRS narrowing and changes in left ventricular volumes. Although these parameters reflect electrical and structural remodeling, they do not fully characterize the interaction between ventricular depolarization, repolarization, and mechanical contraction. The electromechanical window (EMW), defined as the difference between mechanical systole and electrical systole, has emerged as a non-invasive marker of electromechanical coupling. A more negative EMW reflects delayed mechanical contraction relative to ventricular repolarization and has been associated with increased ventricular arrhythmic risk in inherited channelopathies, cardiomyopathies, and acquired repolarization disorders [11–14]. Recent studies have suggested that EMW integrates ventricular depolarization, repolarization, and mechanical systolic function into a single physiological parameter, making it an attractive marker for assessing CRT effectiveness [13, 14].

The influence of different CRT pacing strategies on the electromechanical window has not been systematically investigated. Furthermore, whether EMW reflects reverse remodeling, predicts CRT response, or identifies patients at increased residual arrhythmic risk after CRT remains uncertain. Given the increasing adoption of conduction system pacing and the ongoing debate regarding its advantages over conventional BiV pacing, evaluation of EMW may provide important mechanistic insight into differences between these pacing strategies.

Therefore, this prospective study compared the effects of conventional BiV pacing and LBBAP on the electromechanical window in patients undergoing CRT. We further evaluated the relationship between EMW and reverse ventricular remodeling, CRT response, and device-detected arrhythmic events to explore the potential role of EMW as an integrative physiological marker of CRT effectiveness.

## Methods

This prospective, single-center observational study enrolled 80 consecutive patients with HFrEF who underwent CRT implantation between January 2023 and June 2025. Eligible patients had a left ventricular ejection fraction (LVEF) ≤ 35%, New York Heart Association (NYHA) functional class II–IV symptoms despite guideline-directed medical therapy, and a QRS duration ≥ 130 ms with left bundle branch block morphology or ≥ 150 ms with non-left bundle branch block morphology. Patients with permanent atrial fibrillation and uncontrolled ventricular rates, significant primary valvular heart disease requiring surgical intervention, previous CRT implantation, congenital heart disease, or inadequate echocardiographic imaging windows were excluded.

Because this was an observational study, patients were not randomized. The choice of pacing modality (conventional BiV pacing or LBBAP) was determined according to operator expertise, anatomical feasibility, and successful lead implantation. Conventional BiV pacing was performed using standard right atrial, right ventricular, and coronary sinus left ventricular leads. Left ventricular lead placement was preferentially targeted to the lateral or posterolateral coronary sinus branches whenever anatomically feasible. LBBAP was performed using transseptal lead deployment targeting the left bundle branch area. Conduction system capture was confirmed according to contemporary European Heart Rhythm Association (EHRA) consensus recommendations using a combination of electrocardiographic criteria, including diagnostic QRS morphology transition during threshold testing, paced stimulus-to-peak R-wave time in lead V6 ≤ 90 ms, V6–V1 interpeak interval > 44 ms, and diagnostic QRS morphology transition during programmed stimulation [15].

Following implantation, all patients underwent routine device optimization within four weeks using echocardiographic assessment of atrioventricular and interventricular timing according to institutional practice. Device interrogation was performed at six months to evaluate lead performance, pacing parameters, high atrial rate episodes (HARE), and ventricular arrhythmias, including non-sustained ventricular tachycardia (NSVT).

Comprehensive transthoracic echocardiography with simultaneous 12-lead electrocardiographic recording was performed at baseline and six months using a standardized acquisition protocol. Left ventricular volumes and ejection fraction were calculated using the modified Simpson biplane method. Global longitudinal strain (GLS) was assessed using two-dimensional speckle-tracking echocardiography.

**Fig. 1:**
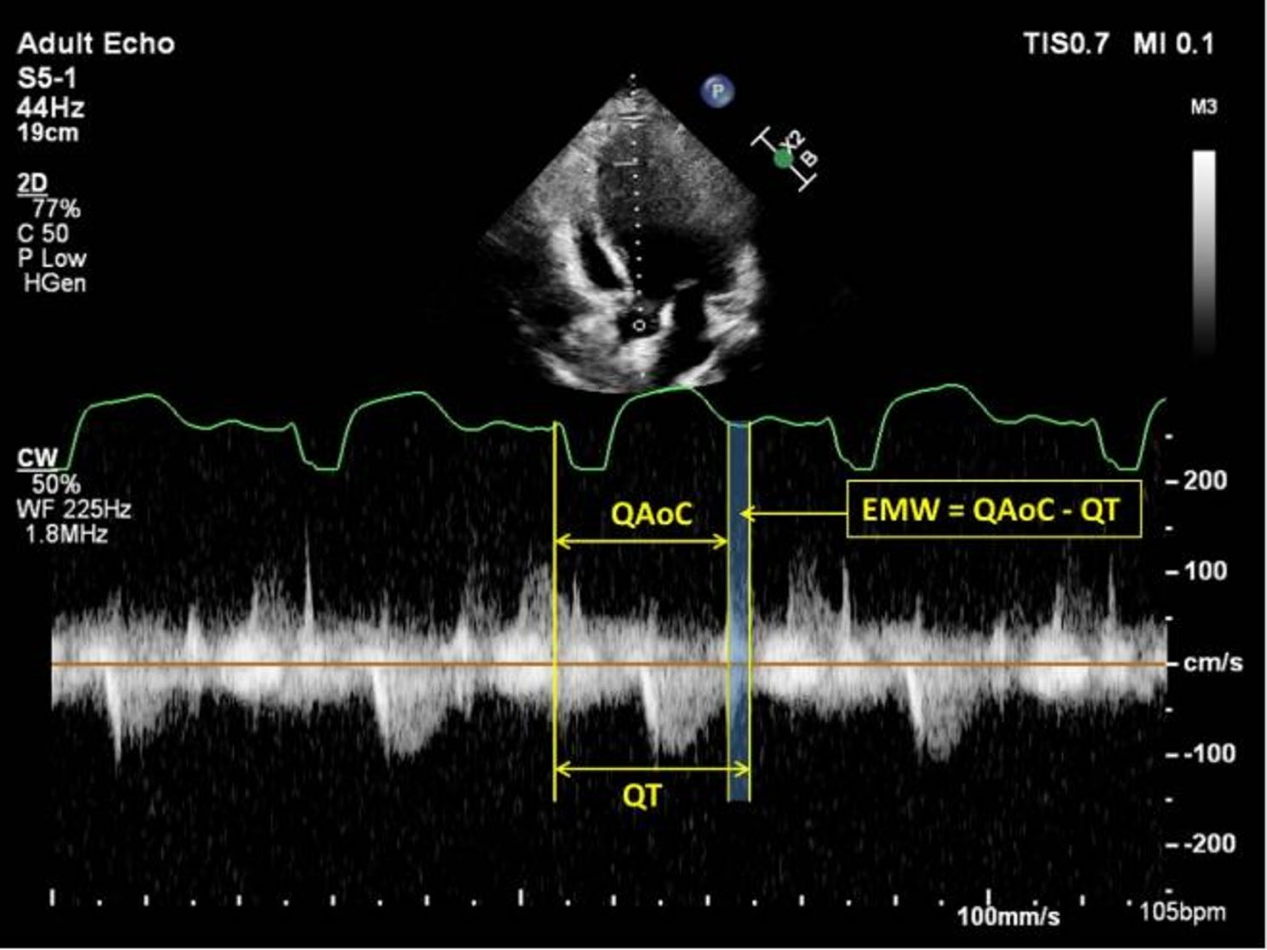
Five-chamber echocardiographic view demonstrating measurement of the electromechanical window (EMW). Mechanical systole (QAoC) was defined as the interval from QRS onset to aortic valve closure (AoC), obtained from continuous-wave Doppler of the left ventricular outflow tract. Electrical systole was defined as the QT interval measured from QRS onset to the end of the T wave on the simultaneously recorded electrocardiogram. EMW was calculated as QAoC minus the QT interval, with negative values indicating electromechanical uncoupling

Mechanical systole was defined as the interval from QRS onset to aortic valve closure measured from continuous-wave Doppler recordings obtained across the left ventricular outflow tract. Electrical systole was defined as the QT interval measured from QRS onset to the end of the T wave on the simultaneously recorded electrocardiogram. EMW was calculated as mechanical systole minus the QT interval (EMW = QAoC – QT, Fig. 1), with negative values indicating electromechanical uncoupling. To minimize beat-to-beat variability, EMW measurements were averaged over three consecutive cardiac cycles obtained during stable sinus rhythm. Blood pressure remained clinically stable during echocardiographic acquisition. QRS duration was measured digitally from the pacing stimulus (or QRS onset in intrinsic rhythm) to the latest offset of the QRS complex in any of the 12 ECG leads using magnified recordings.

The primary endpoint was the change in EMW at six months. Secondary endpoints included changes in QRS duration, LVEF, GLS, left ventricular end-systolic volume (LVESV), CRT response (defined as ≥ 15% reduction in LVESV), device-detected atrial and ventricular arrhythmias, and the relationship between EMW, reverse remodeling, and CRT response.

### Statistical analysis

Statistical analyses were performed using SPSS version 27.0 (IBM Corp., Armonk, NY, USA). Continuous variables are presented as mean ± standard deviation and categorical variables as counts and percentages. Within-group changes from baseline to six months were assessed using paired t-tests or Wilcoxon signed-rank tests as appropriate. Between-group comparisons (BiV vs. LBBAP) were performed using independent t-tests or Mann–Whitney U tests. Categorical variables were compared using the chi-square or Fisher’s exact test.

Correlations between changes in EMW and echocardiographic parameters were evaluated using Pearson or Spearman correlation coefficients. Multivariable linear regression analysis was performed to identify independent predictors of EMW improvement. Receiver operating characteristic (ROC) analysis was used to assess the ability of baseline EMW to predict CRT response. A two-tailed p value < 0.05 was considered statistically significant.

## Results

Forty patients underwent conventional BiV pacing and forty underwent LBBAP. Baseline demographic, clinical, and echocardiographic characteristics are summarized in Table 1. The mean age of the study population was 62 ± 10 years, and 72% were male. Ischemic cardiomyopathy was present in 48% of patients, while baseline comorbidities, heart failure medications, and echocardiographic characteristics were comparable between the two groups. The overall mean LVEF was 27.5 ± 5.5%, mean LVESV was 155 ± 35 mL, QRS duration was 163 ± 19 ms, and EMW was − 37 ± 21 ms.

**Table 1:** Baseline characteristics

Parameter	Overall (*n* = 80)	BiV (*n* = 40)	LBBAP (*n* = 40)	*p* value
Age (years)	62 ± 10	63 ± 9	61 ± 10	0.34
Male sex, n (%)	58 (72%)	30 (75%)	28 (70%)	0.62
Ischemic cardiomyopathy, n (%)	38 (48%)	20 (50%)	18 (45%)	0.65
Hypertension, n (%)	48 (60%)	25 (63%)	23 (58%)	0.64
Diabetes mellitus, n (%)	30 (38%)	16 (40%)	14 (35%)	0.65
NYHA Class III/IV	58 (73%)	30 (75%)	28 (70%)	0.61
ACEI/ARB/ARNI therapy	72 (90%)	36 (90%)	36 (90%)	1.00
Beta-blocker therapy	76 (95%)	38 (95%)	38 (95%)	1.00
Mineralocorticoid receptor antagonist	66 (83%)	33 (83%)	33 (83%)	1.00
SGLT2 inhibitor	54 (68%)	26 (65%)	28 (70%)	0.63
LVEF (%)	27.5 ± 5.5	28 ± 5	27 ± 6	0.41
LVESV (mL)	155 ± 35	154 ± 36	156 ± 34	0.78
GLS (%)	−9.8 ± 2.4	−9.7 ± 2.5	−9.9 ± 2.3	0.74
QRS duration (ms)	163 ± 19	162 ± 18	165 ± 20	0.48
Electromechanical Window (ms)	−37 ± 21	−36 ± 20	−38 ± 22	0.68

Among patients undergoing LBBAP, successful conduction system capture according to EHRA criteria was achieved in 36 of 40 patients (90%) at implantation and was maintained in 34 patients (85%) at six-month follow-up. In the BiV group, the left ventricular lead was positioned in the lateral or posterolateral coronary sinus branch in 32 patients (80%), while the remaining patients had anterolateral or middle cardiac vein positions because of unfavorable coronary venous anatomy.

Six-month follow-up data are presented in Table 2. Both pacing strategies resulted in significant electrical and mechanical improvement; however, these changes were consistently greater in the LBBAP group. QRS duration decreased significantly in both groups, with greater QRS narrowing observed after LBBAP than BiV pacing (42 ± 15 ms vs. 28 ± 13 ms, *p* < 0.001). Likewise, EMW improved significantly in both groups but demonstrated greater normalization following LBBAP (from − 38 ± 22 ms to − 5 ± 18 ms) compared with BiV pacing (from − 36 ± 20 ms to − 18 ± 19 ms; between-group *p* = 0.003). Improvements in LVEF, GLS, and LVESV were also significantly greater in the LBBAP group (Table 2).

**Table 2:** Procedural characteristics and six-month follow-up outcomes

Parameter	Overall	BiV	LBBAP	*p* value
Procedural Characteristics				
Successful conduction system capture (%)	–	–	36 (90%)	–
Conduction system capture at 6 months (%)	–	–	34 (85%)	–
LV lead in lateral/posterolateral vein (%)	–	32 (80%)	–	–
Follow-up Outcomes (6 months)				
QRS duration (ms)	128 ± 17	134 ± 16	123 ± 14	0.002
Change in QRS (ms)	−35 ± 16	−28 ± 13	−42 ± 15	< 0.001
EMW (ms)	−12 ± 19	−18 ± 19	−5 ± 18	0.003
Change in EMW (ms)	+ 26 ± 18	+ 18 ± 16	+ 33 ± 17	0.003
LVEF (%)	36.5 ± 7	35 ± 6	38 ± 7	0.04
Change in LVEF (%)	+ 9 ± 6	+ 7 ± 5	+ 11 ± 6	0.004
GLS (%)	−14.9 ± 3.1	−13.6 ± 2.9	−16.3 ± 3.0	0.001
Change in GLS (%)	−5.1 ± 2.4	−4.1 ± 2.3	−6.2 ± 2.1	0.001
LVESV (mL)	125 ± 32	134 ± 34	116 ± 28	0.02
Change in LVESV (%)	−19 ± 11	−15 ± 10	−22 ± 11	0.01
CRT response (%)	60%	50%	70%	0.048
HARE (%)	31%	40%	22%	0.048
NSVT (%)	25%	35%	15%	0.03

Correlation analysis demonstrated that EMW improvement was significantly associated with indices of reverse remodeling and electrical resynchronization (Table 3). Improvement in EMW correlated moderately with LVESV reduction (*r* = 0.46, 95% CI 0.27–0.61; *p* < 0.001), GLS improvement (*r* = 0.42, 95% CI 0.22–0.58; *p* = 0.002), LVEF improvement (*r* = 0.39, 95% CI 0.18–0.56; *p* = 0.004), and QRS narrowing (*r* = 0.35, 95% CI 0.13–0.53; *p* = 0.01), supporting EMW as an integrated marker of electrical and mechanical recovery.

**Table 3:** Correlation between EMW improvement and markers of electrical and ventricular remodeling

Variable	Correlation coefficient (*r*)	95% CI	*p* value
LVESV reduction	0.46	0.27–0.61	< 0.001
GLS improvement	0.42	0.22–0.58	0.002
LVEF improvement	0.39	0.18–0.56	0.004
QRS narrowing	0.35	0.13–0.53	0.01

Multivariable linear regression analysis identified independent predictors of EMW improvement at six months (Table 4). Greater LVESV reduction (β = 0.34, *p* = 0.001), GLS improvement (β = 0.29, *p* = 0.003), and LBBAP (β = 0.31, *p* = 0.002) independently predicted greater EMW normalization. Baseline EMW also independently predicted subsequent EMW improvement (β=−0.22, *p* = 0.01), while QRS narrowing remained a weaker but significant predictor (β = 0.18, *p* = 0.02). The final model explained 48% of the variability in EMW improvement (R²=0.48, *p* < 0.001).

**Table 4:** Multivariable predictors of EMW normalization

Variable	β coefficient	95% CI	*p* value
LVESV reduction	0.34	0.14–0.54	0.001
GLS improvement	0.29	0.10–0.48	0.003
LBBAP	0.31	0.12–0.50	0.002
Baseline EMW	−0.22	−0.39 to − 0.05	0.01
QRS narrowing	0.18	0.03–0.34	0.02

β = standardized regression coefficient; CI = confidence interval

Device interrogation at six months demonstrated a significantly lower arrhythmic burden among patients treated with LBBAP. High atrial rate episodes occurred in 22% of LBBAP patients compared with 40% of patients receiving BiV pacing (*p* = 0.048), while non-sustained ventricular tachycardia (NSVT) occurred in 15% and 35%, respectively (*p* = 0.03). Persistent EMW negativity at follow-up was associated with a higher incidence of NSVT, irrespective of pacing modality (45% in patients with persistent EMW negativity vs. 18% in those with EMW normalization, *p* = 0.01).

**Fig. 2:**
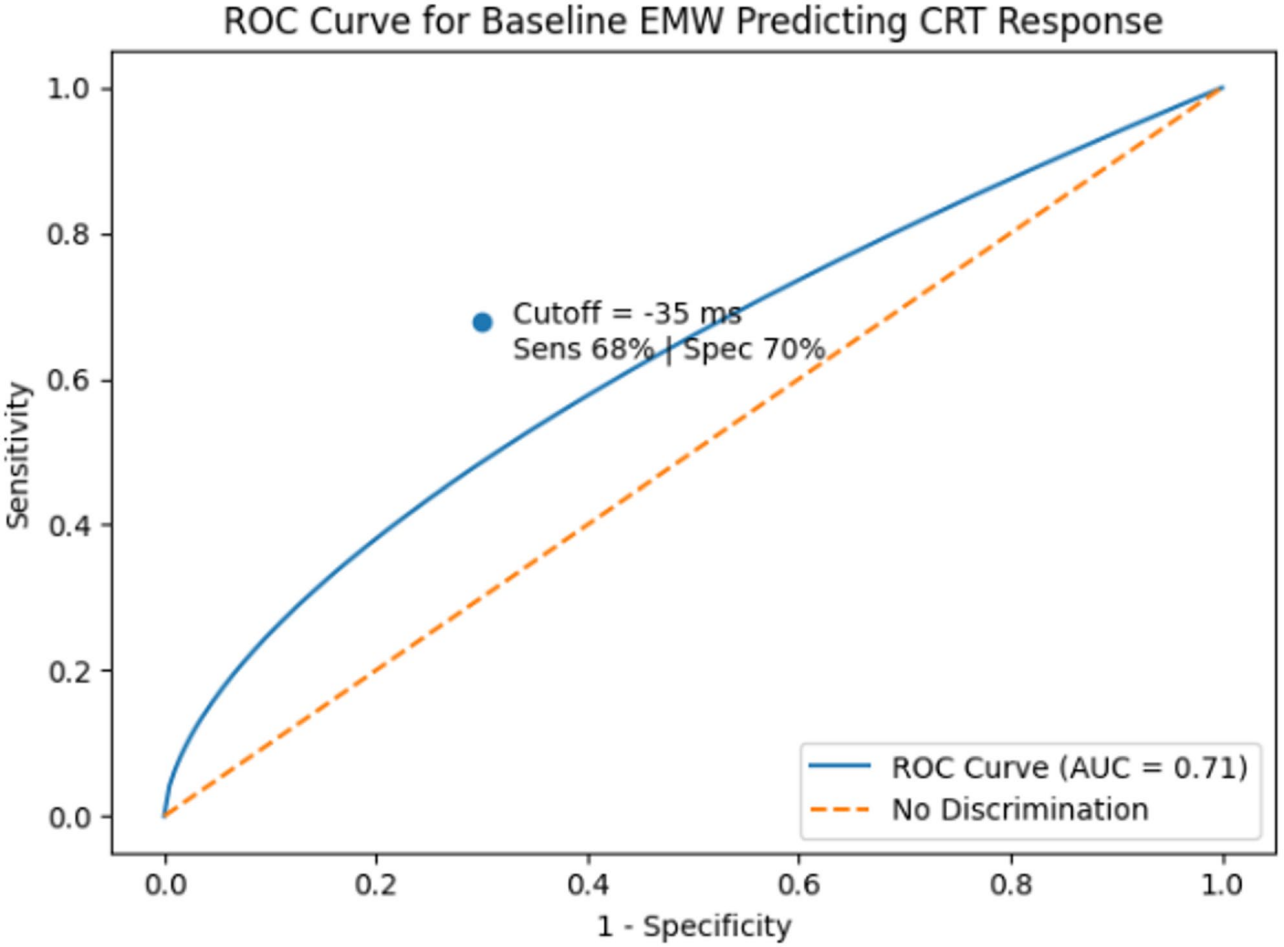
Receiver operating characteristic (ROC) curve showing the predictive value of baseline electromechanical window (EMW) for CRT response (AUC = 0.71). An EMW cutoff of − 35 ms provided 68% sensitivity and 70% specificity

CRT response, defined as a ≥ 15% reduction in LVESV, was observed more frequently in the LBBAP group than in the BiV group (70% vs. 50%, *p* = 0.048). Comparison of responders and non-responders is summarized in Table 5. Responders demonstrated significantly more negative baseline EMW values than non-responders (− 42 ± 21 ms vs. − 29 ± 18 ms, *p* = 0.01), greater EMW normalization (+ 34 ± 18 ms vs. + 16 ± 14 ms, *p* = 0.002), and less residual EMW negativity at follow-up (− 8 ± 17 ms vs. − 19 ± 20 ms, *p* = 0.01). Receiver operating characteristic analysis demonstrated that baseline EMW predicted CRT response with moderate accuracy (AUC 0.71; Fig. 2). An EMW cutoff value of − 35 ms yielded a sensitivity of 68% and specificity of 70% for predicting CRT response.

**Table 5:** Comparison of CRT responders and non-responders

Parameter	CRT Responders (*n* = 48)	Non-Responders (*n* = 32)	*p* value
Age (years)	61 ± 9	63 ± 11	0.34
Male sex (%)	73%	69%	0.68
Baseline LVEF (%)	27 ± 5	28 ± 6	0.37
Baseline LVESV (mL)	160 ± 34	148 ± 36	0.09
Baseline QRS (ms)	166 ± 19	159 ± 17	0.07
Baseline EMW (ms)	−42 ± 21	−29 ± 18	0.01
EMW change at 6 months (ms)	+ 34 ± 18	+ 16 ± 14	0.002
Final EMW (ms)	−8 ± 17	−19 ± 20	0.01
LVEF improvement (%)	+ 13 ± 6	+ 4 ± 3	< 0.001
LVESV reduction (%)	−28 ± 8	−6 ± 5	< 0.001
GLS improvement	−7.2 ± 2.1	−2.4 ± 1.6	< 0.001
NSVT at 6 months (%)	17%	38%	0.03

Compared with non-responders, CRT responders exhibited significantly greater reverse remodeling, with larger improvements in LVEF (+ 13 ± 6% vs. + 4 ± 3%, *p* < 0.001), greater LVESV reduction (− 28 ± 8% vs. − 6 ± 5%, *p* < 0.001), and greater GLS improvement (− 7.2 ± 2.1% vs. − 2.4 ± 1.6%, *p* < 0.001). Non-responders also experienced a significantly higher incidence of NSVT during follow-up (38% vs. 17%, *p* = 0.03), whereas baseline demographic and clinical characteristics were otherwise comparable between the two groups.

## Discussion

The present study demonstrates that both conventional BiV pacing and LBBAP were associated with significant improvement in electromechanical coupling, reflected by a shift of EMW toward less negative values. However, patients undergoing LBBAP exhibited greater EMW normalization, more pronounced reverse remodeling, superior electrical resynchronization, and a lower burden of device-detected atrial and ventricular arrhythmias at six months. These findings suggest that successful physiological conduction system pacing may provide more effective restoration of electromechanical synchrony than conventional BiV pacing, although these observations should be interpreted within the context of our nonrandomized study design.

Cardiac resynchronization therapy has traditionally been delivered using BiV pacing, which has consistently improved symptoms, ventricular function, and survival in appropriately selected patients with heart failure [1–3]. Conduction system pacing, particularly LBBAP, has emerged as an alternative strategy because it restores ventricular activation through the native His–Purkinje conduction system. Early observational studies suggested superior electrical synchrony and reverse remodeling with LBBAP compared with BiV pacing [6, 7]. However, more recent randomized controlled trials have demonstrated that the clinical and echocardiographic benefits of LBBAP are largely dependent on successful conduction system capture, and that LBBAP is generally comparable to conventional BiV pacing when physiological conduction is effectively achieved [8–10]. In the present study, successful conduction system capture was achieved in 90% of patients at implantation and maintained in 85% at six-month follow-up, which may partly explain the greater electromechanical recovery observed in the LBBAP group.

The electromechanical window represents an integrated measure of ventricular depolarization, repolarization, and mechanical systole rather than isolated electrical or mechanical function. Negative EMW values have previously been associated with ventricular arrhythmias in inherited channelopathies, cardiomyopathies, and acquired repolarization abnormalities [11–14]. The present study extends these observations to patients undergoing CRT by demonstrating that EMW also reflects the degree of electromechanical recovery following resynchronization therapy. Importantly, EMW improvement correlated with QRS narrowing, improvement in GLS, LVEF, and LV reverse remodeling, suggesting that EMW integrates multiple aspects of CRT response into a single physiological parameter.

Multivariable analysis further supports this concept. After adjustment for electrical and structural variables, greater LVESV reduction, improvement in GLS, baseline EMW, and LBBAP independently predicted EMW normalization. These findings indicate that EMW is influenced by both electrical resynchronization and mechanical recovery rather than either process alone. Accordingly, EMW may represent an integrative physiological marker of CRT effectiveness that complements conventional electrical and echocardiographic measures.

An important observation of the present study is the relationship between EMW and CRT response. Patients who demonstrated reverse remodeling exhibited significantly more negative baseline EMW values together with greater EMW normalization after CRT. Baseline EMW predicted CRT response with moderate discriminatory ability (AUC 0.71), suggesting that greater baseline electromechanical uncoupling may identify patients with greater potential for recovery following successful resynchronization. The overall CRT response rate was higher in the LBBAP group than in the BiV group (70% vs. 50%). Although the response rate in the BiV arm appears lower than that reported in some contemporary randomized trials, including the CSP-SYNC and LEFT-BUNDLE-CRT studies [9, 10], differences in patient selection, study design, operator experience, left ventricular lead position, and optimization protocols may account for this variation. Moreover, recent randomized evidence suggests that the clinical advantage of LBBAP over BiV pacing is most evident when successful conduction system capture is achieved, emphasizing the importance of procedural success rather than pacing modality alone. In our study, successful conduction system capture was confirmed in 90% of patients at implantation and maintained in 85% at six-month follow-up, which may have contributed to the favorable remodeling observed in the LBBAP group. Although these findings require external validation, they suggest that EMW may complement established predictors of CRT response and potentially improve patient selection and post-implant assessment in the future.

The association between EMW and arrhythmic burden is another noteworthy finding. Patients treated with BiV pacing demonstrated a higher incidence of HARE and NSVT than those undergoing LBBAP. Furthermore, persistent EMW negativity after CRT was associated with a significantly greater incidence of NSVT (45% vs. 18%), irrespective of pacing modality, while CRT non-responders also experienced more ventricular arrhythmias than responders. These findings support the concept that incomplete restoration of electromechanical synchrony may reflect persistent dispersion of ventricular repolarization and mechanical dyssynchrony, creating an arrhythmogenic substrate that predisposes to ventricular arrhythmias, whereas physiological conduction system pacing may reduce repolarization heterogeneity and improve electrical stability [16].

Samways et al. demonstrated that epicardial BiV pacing may increase repolarization dispersion compared with conduction system pacing, providing a potential mechanistic explanation for the greater EMW normalization observed with LBBAP [17]. Because EMW integrates electrical repolarization and mechanical systole, improved physiological activation through the His–Purkinje system may reduce repolarization heterogeneity and enhance electromechanical stability. Consistent with this concept, the recently published ICLAS study also demonstrated lower ventricular arrhythmia burden and favorable clinical outcomes with LBBAP compared with conventional BiV pacing [8]. Although our study was not powered to evaluate long-term arrhythmic endpoints, the observed association between persistent EMW negativity and NSVT supports the hypothesis that EMW may serve as a marker of residual electromechanical instability following CRT.

Despite these encouraging findings, our results should be interpreted cautiously. Recent randomized studies comparing LBBAP with BiV pacing have demonstrated that clinical superiority is not universal and depends largely on successful conduction system engagement, patient selection, and lead implantation technique [9, 10]. Therefore, our findings should not be interpreted as evidence that LBBAP is universally superior to conventional CRT. Rather, they suggest that when physiological conduction system capture is achieved, greater restoration of electromechanical coupling may occur.

From a clinical perspective, EMW has several potential applications. Unlike QRS duration or ventricular volumes alone, EMW integrates electrical activation, repolarization, and mechanical contraction into a single physiological measure. Consequently, EMW may provide incremental value for comparing pacing strategies, identifying patients most likely to respond to CRT, assessing residual arrhythmic risk after implantation, and potentially guiding post-implant optimization. However, these applications remain investigational and larger multicenter randomized studies with standardized EMW measurement protocols, longer follow-up, and adjudicated clinical endpoints are required to determine whether EMW-guided selection or optimization of CRT improves long-term remodeling, arrhythmic outcomes, and survival.

## Limitations

This study has several limitations:

- Single-center, nonrandomized observational design: The choice of pacing modality was based on operator expertise and anatomical feasibility rather than randomization, introducing the potential for selection bias despite comparable baseline characteristics.
- Modest sample size and short follow-up: The relatively small cohort and 6-month follow-up limited assessment of long-term clinical outcomes, including mortality, heart failure hospitalization, sustained ventricular arrhythmias, implantable cardioverter-defibrillator (ICD) therapies, and sudden cardiac death. The study was not powered for these hard clinical endpoints.
- EMW measurement limitations: Although EMW measurements were obtained using a standardized protocol with simultaneous ECG recording and averaged over three consecutive cardiac cycles, formal interobserver and intraobserver reproducibility analyses were not performed. In addition, mechanical systole was determined using aortic valve closure, which is influenced by loading conditions such as blood pressure.
- Limited imaging assessment: Advanced imaging techniques, including tissue Doppler imaging and cardiac magnetic resonance imaging, were not performed. Consequently, the relationship between EMW, myocardial scar burden, and mechanical dyssynchrony could not be evaluated.
- Generalizability: The findings require validation in larger multicenter randomized studies before EMW can be recommended as a routine marker for CRT selection, optimization, or risk stratification.

## Clinical implications

The present study suggests that the electromechanical window may serve as an integrative physiological marker linking electrical resynchronization, reverse remodeling, and arrhythmic vulnerability in patients undergoing CRT. Beyond conventional assessment using QRS duration and ventricular remodeling, EMW may provide incremental information for evaluating pacing strategies, identifying patients more likely to respond to CRT, and recognizing persistent electromechanical instability following device implantation. Prospective multicenter studies are required before EMW can be incorporated into routine clinical practice.

## Conclusion

In this prospective observational study, LBBAP was associated with greater normalization of EMW, more pronounced reverse ventricular remodeling, and a lower burden of device-detected arrhythmias compared with conventional BiV pacing. Improvement in EMW correlated with electrical resynchronization, myocardial functional recovery, and reverse remodeling, while baseline EMW demonstrated moderate ability to predict CRT response.

These findings suggest that EMW may serve as a novel integrative physiological marker of CRT effectiveness, with potential applications in evaluating pacing strategies, predicting response to therapy, and identifying patients at persistent arrhythmic risk. However, given the observational design and modest sample size of this study, these results should be considered hypothesis-generating. Larger multicenter randomized studies with longer follow-up are warranted to validate the clinical utility of EMW and to determine whether EMW-guided CRT optimization can improve long-term clinical outcomes.

## Data Availability

No datasets were generated or analysed during the current study.

## Declarations

### Declarations of generative AI and AI-assisted technologies in the manuscript preparation process

During the preparation of this work the author(s) used ChatGPT in order to paraphrase few sentences. After using this tool/service, the author(s) reviewed and edited the content as needed and take(s) full responsibility for the content of the published article.
